# Human platelet lysate as a fetal bovine serum substitute improves human adipose-derived stromal cell culture for future cardiac repair applications

**DOI:** 10.1007/s00441-012-1360-5

**Published:** 2012-03-07

**Authors:** B. A. Naaijkens, H. W. M. Niessen, H-J. Prins, P. A. J. Krijnen, T. J. A. Kokhuis, N. de Jong, V. W. M. van Hinsbergh, O. Kamp, M. N. Helder, R. J. P. Musters, A. van Dijk, L. J. M. Juffermans

**Affiliations:** 1 Department of Pathology, VU University Medical Center, De Boelelaan 1117, 1081 HV Amsterdam, The Netherlands; 2 Department of Cardiac Surgery, VU University Medical Center, Amsterdam, The Netherlands; 3 Department of Oral & Maxillofacial Surgery, VU University Medical Center, Amsterdam, The Netherlands; 4 Department of Physiology, VU University Medical Center, Amsterdam, The Netherlands; 5 Department of Orthopaedics, VU University Medical Center, Amsterdam, The Netherlands; 6 Department of Cardiology, VU University Medical Center, Amsterdam, The Netherlands; 7 Institute of Cardiovascular Research (ICaR-VU), VU University Medical Center, Amsterdam, The Netherlands; 8 Oral Cell Biology, Academic Centre for Dentistry, Amsterdam, The Netherlands; 9 Interuniversity Cardiology Institute of the Netherlands (ICIN), Utrecht, The Netherlands; 10 Department of Biomedical Engineering, Erasmus MC, Rotterdam, The Netherlands; 11 Department of Physics of Fluids, University of Twente, Enschede, The Netherlands; 12 Research Institute MOVE, Amsterdam, The Netherlands

**Keywords:** Adipose-derived stromal cells, Platelet lysate, Cardiac differentiation, Human

## Abstract

Adipose-derived stromal cells (ASC) are promising candidates for cell therapy, for example to treat myocardial infarction. Commonly, fetal bovine serum (FBS) is used in ASC culturing. However, FBS has several disadvantages. Its effects differ between batches and, when applied clinically, transmission of pathogens and antibody development against FBS are possible. In this study, we investigated whether FBS can be substituted by human platelet lysate (PL) in ASC culture, without affecting functional capacities particularly important for cardiac repair application of ASC. We found that PL-cultured ASC had a significant 3-fold increased proliferation rate and a significantly higher attachment to tissue culture plastic as well as to endothelial cells compared with FBS-cultured ASC. PL-cultured ASC remained a significant 25% smaller than FBS-cultured ASC. Both showed a comparable surface marker profile, with the exception of significantly higher levels of CD73, CD90, and CD166 on PL-cultured ASC. PL-cultured ASC showed a significantly higher migration rate compared with FBS-cultured ASC in a transwell assay. Finally, FBS- and PL-cultured ASC had a similar high capacity to differentiate towards cardiomyocytes. In conclusion, this study showed that culturing ASC is more favorable in PL-supplemented medium compared with FBS-supplemented medium.

## Introduction

Adipose-derived stromal cells (ASC) are promising candidates for cell therapy, as they are mesenchymal multipotent cells that have been shown to be able to differentiate towards osteoblasts, chondrocytes, neurons, adipocytes, skeletal myocytes, and cardiomyocytes (Jang et al. 2010; Rangappa et al. 2003; Varma et al. 2007; Zuk et al. 2002; van Dijk et al. 2011). ASC can be obtained via a minimally invasive method such as liposuction, resulting in minimal patient discomfort and low patient risk. Furthermore, adipose tissue provides up to 100 times more mesenchymal stromal cells than bone marrow (Katz et al. 2005; Nakagami et al. 2006; Tholpady et al. 2006; Zhu et al. 2008; Varma et al. 2007), and in contrast to embryonic stem cells, ASC can be used autologously (Nakagami et al. 2006; Tholpady et al. 2006). It has been shown that during culture ASC retain their proliferation capacity, cell surface marker profile, multipotency, and expression of transcription factors that regulate the homeostasis of stem cells (Zhu et al. 2008). Therefore, ASC have gained great appeal in regenerative medicine, for example to treat myocardial infarction.

Isolation and culture expansion of ASC is commonly performed in media supplemented with fetal bovine serum (FBS). However, for clinical applications, the use of reagents of animal sources is undesirable, due to potential transmission of animal pathogens and the risk that antibodies against FBS are developed, leading to rejection of the transfused cells (Halme and Kessler 2006; Heiskanen et al. 2007; Horn et al. 2010). Furthermore, high variability between FBS batches hampers reproducibility and consistency between results from cell culture experiments obtained in different laboratories (Halme and Kessler 2006). Another disadvantage of culturing in FBS-supplemented medium is that it increases cell size, which can result in obstruction of small capillaries when injected intravenously (Zhu et al. 2008; Bieback et al. 2010; Blande et al. 2009; Fischer et al. 2009). Regulatory guidelines for clinical usage of cellular products aim to replace FBS by human cell culture supplements (Halme and Kessler 2006). Human reagents, such as human platelet lysate (PL), could thus enhance safety, quality and reproducibility of in vitro expanded ASC. PL is produced from highly concentrated human platelets in plasma that is of clinical grade, and allows stem cells to be expanded under good manufacturing practice (GMP) conditions (Kakudo et al. 2008; Kocaoemer et al. 2007; Blande et al. 2009). PL contains, amongst others, platelet-derived growth factor, basic fibroblast growth factor, vascular endothelial growth factor, insulin growth factor-1, and transforming growth factor-β that in culture serve as growth factors for cells (Doucet et al. 2005; Eppley et al. 2004; Shih et al. 2010). PL can be produced from a pool of donors which allows in advance production of a GMP-approved product without extensive testing (Ploderl et al. 2010; Shih et al. 2010; Prins et al. 2009). Several characteristics of human PL-cultured ASC have been investigated previously, indicating enhanced proliferation rate and a similar cell surface marker profile (Blande et al. 2009; Kakudo et al. 2008; Kocaoemer et al. 2007; Bieback et al. 2010; Crespo-Diaz et al. 2011; Shih et al. 2010; Castegnaro et al. 2011). Before translating PL culturing to clinical use, more characteristics of PL-cultured ASC need to be analyzed. In this study, we investigated several in vitro characteristics particularly important for the cardiac repair potential of human PL-cultured ASC, including proliferation, attachment, cell size, migration, and differentiation towards cardiomyocytes.

## Materials and methods

### Adipose tissue donors

Human subcutaneous adipose tissue samples from elective plastic surgery were obtained from the department of Plastic Surgery (Tergooi Hospital in Hilversum, the Netherlands) according to hospital guidelines after written informed consent. Adipose tissue was harvested from the abdomen after resection. In this study 11 female donors (age range: 32–67 years, mean: 51 years) were included. This study complied with the principles of the Declaration of Helsinki.

### Isolation of stromal vascular fraction of adipose tissue

After surgery, adipose tissue was stored in sterile phosphate-buffered saline (PBS; B. Braun, Melsungen, Germany) at 4ºC overnight and processed within 24 h, as described previously (Varma et al. 2007). In brief, adipose tissue was minced using a surgical scalpel. After washing with PBS, the material was enzymatically digested with 0.1% collagenase A (Roche Diagnostics, Mannheim, Germany) in PBS containing 1% bovine serum albumin (BSA; Roche Diagnostics) under continuous shaking conditions for 45 min at 37ºC. After Ficoll density centrifugation (Lymphoprep, 1,000 *g*, 20 min, ρ = 1.077 g/ml, osmolarity 280 ± 15 mOsm; Axis-Shield, Oslo, Norway), the cell containing interface was harvested, counted, frozen using recovery cell culture freezing medium (Gibco, Invitrogen, CA, USA), and subsequently stored in liquid nitrogen.

### Platelet lysate

Platelet concentrates were produced as described in the article of de Korte et al.^2006^. In short, whole blood was collected under standardized conditions in a CPD/SAGM system (Fresenius, Emmer-Compascuum, The Netherlands), stored overnight at room temperature and processed into plasma, buffycoat, and leukocyte-reduced red cell concentrate in SAGM. Five buffycoats were pooled with plasma and processed into a leukocyte-reduced platelet concentrate. No stabilizers were used in this protocol (de Korte et al. 2006). These platelet concentrates were obtained from the blood transfusion service (Department of Hematology, VU University Medical Center, Amsterdam, The Netherlands) and contained approximately 1 × 10^9^ platelets per ml (Prins et al. 2009). PL was obtained by lysing the platelet concentrates by temperature shock at −80ºC. Before usage, PL was thawed and centrifuged at 2,000 *g* for 10 min to remove remaining platelet fragments. After centrifugation, PL was stored at 4ºC and used for up to 1 week.

### ASC culture

In all experiments, early passage ASC (p2-3) were used. Cells from the stromal vascular fraction of adipose tissue were seeded at 100,000 cells/cm^2^ and cultured in low glucose Dulbecco’s modified Eagle’s medium (DMEM; Gibco) containing 100 U/ml penicillin, 100 μg/ml streptomycin (both Gibco) and either 10% FBS (Hyclone, South Logan, USA; selected batch for proliferation and differentiation towards cardiomyocytes) or 5% PL and 10 U/ml heparin (Leo Pharma, Amsterdam, The Netherlands), in a humidified atmosphere of 5% CO_2_ at 37ºC. Media were changed twice a week. When near confluent (90%), cells were detached with 0.5 mM EDTA/0.05% trypsin (Gibco) and replated. Phase-contrast light microscopy was used to determine morphology and size of FBS- and PL-cultured ASC.

### Proliferation

To assess population doubling time, ASC were seeded at 2,500 cells/cm^2^ and cultured in medium supplemented with either FBS or PL. After 24 h incubation, unattached cells were removed by washing with PBS. When ASC reached near confluence, cells were replated and the number of cells and days were counted. Population doubling times were calculated as described previously (Jurgens et al. 2008). For this, the following formula was used: days in exponential phase/[(log N2 – log N1)/log2], in which N1 represents the number of ASC at the beginning of the exponential growing phase, and N2 the number of ASC at the end of the exponential growing phase.

### Attachment assay to tissue culture plastic

To investigate differences in rate of attachment of ASC to tissue culture plastic of FBS- or PL-cultured ASC, ASC were seeded at a density of 5,000 cells/cm^2^ in DMEM (supplemented with penicillin, streptomycin, and heparin) without FBS or PL. Cells were allowed to attach in a humidified incubator as described previously (van Dijk et al. 2008a). At distinct times (5, 10, 20, 30, 60, 120, and 180 min, and 24 h) unattached cells were removed by washing with PBS. The number of attached cells was quantified using CyQuant Cell Proliferation Assay Kit (Invitrogen) according to the manufacturer’s protocol.

### Attachment to endothelial cells

To investigate the attachment rate of FBS- and PL-cultured ASC to endothelial cells, human umbilical cord endothelial cells (HUVEC) were obtained, isolated, and cultured as described previously (Juffermans et al. 2009), compliant with the principles of the Declaration of Helsinki. HUVEC were seeded at a high density (50,000 cells/cm^2^) at day 1 in a gelatin (1%; BioRad, The Netherlands)-coated 48-well plate. By day 3, a dense monolayer had formed and ASC were seeded on top of the HUVEC monolayer at a density of 20,000 cells/cm^2^ in Medium 199 (M199; Cambrex, Amsterdam, The Netherlands), supplemented with penicillin, streptomycin, heparin, and 1% human serum albumin (Sanquin, Amsterdam, The Netherlands) without FBS or PL. ASC were allowed to attach in a humified incubator, and at 10 and 60 min, unattached cells were removed by washing with PBS. All cells were subsequently trypsinized, washed with PBS, centrifuged at 600 *g* for 5 min, resuspended in 50 μl FACS buffer (PBS containing 1% BSA and 0.05% sodiumazide) and incubated with anti-CD90-Phycoerythrin (PE) antibody (1:25; BD Biosciences, San José, CA, USA) for 30 min. The percentage of attached ASC was determined by the percentage of CD90-positive cells, as HUVEC are CD90 negative, using fluorescence-activated cell sorting (FACS) Calibur flow cytometer (BD Biosciences) analysis with CellQuest-pro software (BD Biosciences).

### Cell size analysis

Mean size (in μm) of FBS- and PL-cultured ASC was analyzed using the Millipore Scepter cell counter. Cells were trypsinized, washed with PBS, and measured using 60-μm scepter tips. In addition, light scattering properties (forward scatter) were studied using FACS analysis, in which the identical instrument settings were used for FBS- and PL-cultured ASC.

### Flow cytometry analysis

Culture-expanded ASC were analyzed for their cell surface marker profile using FACS analysis as described previously (Varma et al. 2007). All monoclonal antibodies (mAbs) used were of the IgG1 type. Fluorescein isothiocyanate (FITC) conjugated mAbs were used against CD31 (1:10), CD34 (1:25), CD90 (1:25), HLA-ABC (1:20), HLA-DR (1:20), and Lin1 (1:20) (all from BD Biosciences), and CD166 (1:10; RDI, Flanders, NJ, USA). PE-conjugated mAbs were used against CD29 (1:25), CD45 (1:20), CD54 (1:20), CD73 (1:20), CD106 (1:25), and CD117 (1:20) (all from BD Biosciences), and CD105 (1:25, Caltag; Invitrogen, Carlsbad, CA, USA). Nonspecific fluorescence was determined by FITC and PE-conjugated isotype controls (both 1:20; BD Biosciences).

### Transwell assay

Transwell assays were performed in 6.4-mm, 8-μm pore size, fibronectin-coated transwell inserts (Falcon). Cells were trypsinized, and washed with PBS to remove all FBS and PL. Subsequently 50,000 ASC were resuspended in 500 μl DMEM supplemented with penicillin, streptomycin, heparin, and 0.25% human serum albumin, and seeded into the upper compartment of the transwell. As a stimulus, 2% FBS + 1% PL were added to the lower compartment in 1.5 ml DMEM with 0.25% human serum albumin. After 4 h incubation at 37°C, the transwell membrane was washed with PBS and fixed in 4% formaldehyde in PBS for 10 min at room temperature. ASC were stained with DAPI (1:1,000; Invitrogen) to visualize the nuclei. Subsequently, membranes were cut from the insert and mounted onto glass slides using Vectashield (Vector Laboratories, Burlingame, CA, USA). Five fields of view (0.57 μm^2^) were analyzed using fluorescence microscopy. The number of ASC was counted as nuclei visible on the upper and lower side of the membrane (Marianas III, Denver, CO, USA) with a ×40 objective (Zeiss, Germany). The percentage of migrated cells was determined by the number of cells at the lower side / the number of cells at the upper and lower side × 100%.

### Cardiomyocyte differentiation

ASC differentiation towards cardiomyocytes was determined as described previously (van Dijk et al. 2008b). In short, ASC were seeded at 2,500 cells/cm^2^ in a 6-well plate and in chamberslides (Nalge Nunc International, Naperville, IL, USA) coated with 0.12 μg/cm laminin (Roche) in PBS. When near confluence was reached, normal culture medium was replaced by DMEM with penicillin and streptomycin and 1% ITS + Premix (BD Biosciences) supplemented with either 15% FBS or 7.5% PL and heparin. Next, cells were stimulated with 5-aza-2-deoxycytidin (9 μM, Fluka; Sigma Aldrich, St Louis, MO, USA) for 24 h. Control cells were not stimulated with 5-aza-2-deoxycytidin and were cultured in normal culture medium. ASC from the 6-well plate were harvested 10 days, 21 days, and 12 weeks after stimulation and spinned onto microscope slides by centrifugation, at a density of 10,000 cells per slide, for 5 min at 28 *g* (Shandon cytospin 3; Thermo Scientific, Waltman, USA). These cytospin slides were air-dried overnight, and fixed with acetone (100%) for 10 min. ASC on the chamberslides were washed with PBS and fixed with acetone (100%) at 10 and 21 days after stimulation.

### Immunohistochemistry

To analyze and quantify markers present in stimulated ASC, cytospin slides were incubated with mouse mAbs against human desmin (1:75; Sigma), troponin T (1:500; Sigma), α-actinin (1:300; Sigma) and myosin light chain-2α (MLC-2α; 1:100, Synaptic Systems, Göttingen, Germany), as previously described by van Dijk et al. (2008b), and also with rabbit polyclonal Ab against human Connexin43 (1:250, Cx43; Abcam) in PBS containing 1% BSA for 90 min at room temperature. In addition, ASC on chamberslides were incubated with anti-Cx43 (1:250) in PBS containing 1% BSA for 90 min at room temperature. Next, all slides were washed with PBS, Cx43 slides were incubated with biotin-conjugated swine-anti-rabbit antibodies (1:300; Dako Cytomation, Glostrup, Denmark), and for the other markers, slides were incubated with biotin-conjugated rabbit-anti-mouse antibodies (1:300; Dako) for 60 min at room temperature. Subsequently, all slides were washed with PBS and incubated with streptavidin-horseradish peroxidase (1:300; Dako) for 60 min at room temperature. Staining was visualized by using 3-amino-9-ethylcarbazole (AEC; Invitrogen). Next, slides were counterstained with hematoxylin and covered. Negative control slides were incubated with PBS instead of the primary antibody. For quantification, 100 cells per slide were scored blindly.

To determine the putative cardiomyocyte specificity of Cx43, human skeletal and cardiac muscle paraffin-embedded tissues were sectioned and deparaffinized as previously described (Baidoshvili et al. 2006). After incubation with 0.3% H_2_O_2_ in methanol for 30 min, the slides were stained with Cx43 (1:250; Abcam) in PBS containing 1% BSA for 90 min at room temperature. Next, slides were washed with PBS and incubated with EnVision-HRP (1:200; DakoCytomation). Staining was visualized using EnVision-diaminobenzidin (DakoCytomation), counterstained with hematoxilin and covered. Control slides were incubated with PBS instead of the primary antibody.

### Statistics

Statistics were performed with GraphPad Prism 4. A Student’s *t* test or one-way ANOVA was used if scores were distributed normally. If not, a Mann–Whitney test or Kruskall–Wallis test was used for analysis. Bonferroni’s was used as post hoc test. A *p* value smaller than 0.05 was considered statistically significant. Values are shown as mean ± standard deviation.

## Results

### Proliferation

**Fig. 1:**
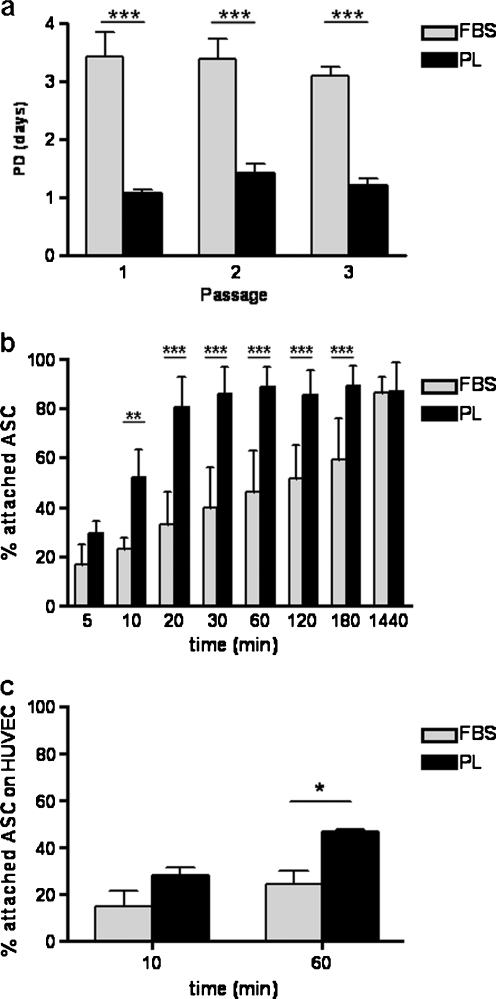
PL-cultured ASC have a short population doubling time and a fast attachment rate. **a** Population doubling time (PD, in days) shown for FBS- and PL-cultured ASC from passages 1–3. *Bars* means ± SD. FBS: *n* = 6, PL: *n* = 5; ***p  < 0.001. **b** Attachment rate of FBS- and PL-cultured cells to tissue culture plastic, shown as percentage of seeded cells at 5, 10, 20, 30, 60, 120, and 180 min and 24 h (1,440 min). *Bars* means ± SD. FBS: *n* = 6, PL: *n* = 5; **p  < 0.01, ***p  < 0.001. **c** Attachment rate of FBS- and PL-cultured ASC to HUVEC, shown as a percentage of seeded cells at 10 and 60 min. *Bars* means ± SD. *n* = 4; *p  < 0.05

To compare the effects of FBS- and PL-supplemented medium on ASC proliferation, population doubling time per passage was calculated (*n* = 5). Multiple FBS batches were tested and one was selected for optimal proliferation and differentiation towards cardiomyocytes. PL-cultured ASC had a doubling time varying between 1.1 ± 0.1 and 1.4 ± 0.2 days in passages 1–3, whereas FBS-cultured cells had a considerably slower doubling time varying between 3.1 ± 0.2 and 3.4 ± 0.4 days (Fig. 1a; *p* < 0.001). In addition, PL-cultured ASC could be cultured up to passage 10 without a significant growth decline (data not shown). Thus, PL significantly enhanced proliferation rate compared with FBS in ASC culture.

### Attachment to tissue culture plastic

The attachment of FBS- or PL-cultured ASC to tissue culture plastic was studied over time (from 5 min up to 24 h, *n* = 5). As depicted in Fig. 1b, after 10 min significantly more PL-cultured ASC had already adhered to the plates compared with FBS-cultured ASC (52.3 ± 11.1 vs. 23.1 ± 4.8%, respectively; *p* < 0.01). Also, at all following time points, significantly more PL-cultured ASC adhered compared with FBS-cultured ASC. In FBS-cultured ASC: adherence increased gradually over time up to 59.2 ± 17.3% after 180 min. In contrast, PL-cultured ASC had already reached 80.4 ± 12.2% adherence after 20 min, which increased to 89.1 ± 8.0% at 180 min. After 24 h, no differences were observed between FBS- and PL-cultured ASC, showing that culturing in PL-supplemented medium primarily enhances early attachment of ASC.

### Attachment to endothelial cells

After intravenous ASC injection, ASC have to attach to endothelial cells prior to extravasation. Therefore, we now demonstrated that FBS- and PL-cultured ASC were capable of attaching to endothelial cells. Attachment of FBS- and PL-cultured ASC to endothelial cells was studied at 10 and 60 min after seeding (*n* = 4), since, in the plastic attachment assay, at 10 min the first significant difference was observed, and at 60 min PL-cultured ASC reached a plateau. As shown in Fig. 1c, both FBS- and PL-cultured ASC were able to adhere to endothelial cells, with a significant difference after 60 min (FBS: 28.0 ± 7.1% vs. PL: 46.7 ± 1.9%, *p* < 0.05).

### Cell size

**Fig. 2:**
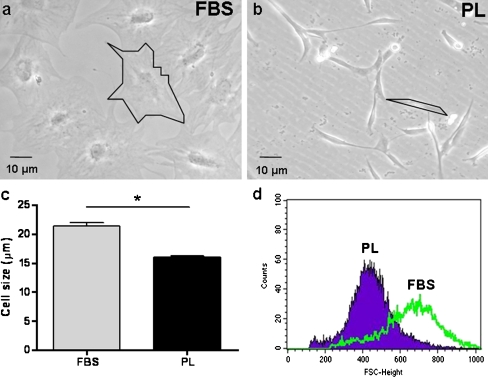
Culture in PL-supplemented medium results in smaller cell size. Differences in cell size shown by phase-contrast light microscopy images. The *black lines* encircle one cell in FBS-cultured ASC (**a**) and PL-cultured ASC (**b**). PL-cultured ASC have a smaller cell size (16.0 ± 0.2µm) compared with FBS-cultured ASC (21.4 ± 0.5µm). *Bars* means + SD. *n *= 5; *p < 0.05 (**c**). Flow cytometry analysis of forward scatter (FSC) of FBS- and PL-cultured ASC showing a more heterogeneous size distribution for FBS-cultured ASC (**d**)

During the proliferation of FBS- and PL-cultured ASC, marked differences in morphology and size were observed in phase-contrast light microscopy images (Fig. 2a, b). FBS-cultured ASC appeared larger and more stretched compared with PL-cultured ASC. This was confirmed using the Millipore Scepter cell counter (*n* = 5), showing that FBS-cultured ASC had a mean diameter of 21.4 ± 0.5 μm and PL-cultured ASC of 16.0 ± 0.2 μm (*p* < 0.05; Fig. 2c). Using a flow cytometry assay and analyzing the forward scatter, this smaller cell size was confirmed and also showed that PL-cultured ASC had a narrower distribution of cell size compared with FBS-cultured ASC (Fig. 2d).

### Cell surface markers

**Fig. 3:**
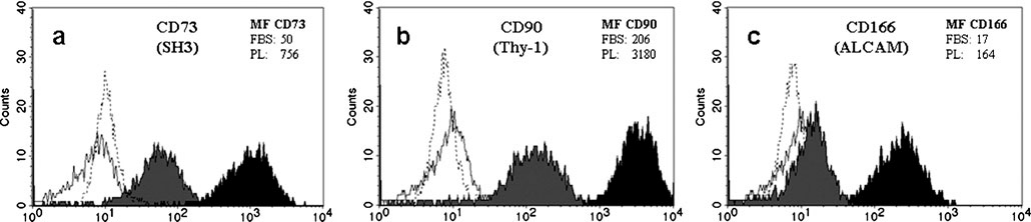
PL-cultured ASC have higher levels of CD73, CD90, and CD166. Flow cytometry histograms of mAb binding to FBS- (*gray*) and PL-cultured ASC (*black*). Overlays of one representative donor are shown for CD73 (**a**), CD90 (**b**), and CD166 (**c**), with its IgG1-isotype controls (*solid line*: FBS-cultured ASC; *dashed line*: PL-cultured ASC)

To determine whether culturing in the two different media affected ASC phenotype, cell surface markers were characterized (*n* = 4). Both FBS- and PL-cultured ASC stained positive for stem cell-associated markers CD29 (β1 integrin), CD34, CD54 (ICAM-1), CD73 (SH3), CD90 (Thy-1), CD105 (SH2), CD166 (ALCAM), and HLA-ABC (see Table 1). No staining was found for hematopoietic or leukocyte markers Lin1, CD45, CD117 (c- kit), and HLA-DR, nor for endothelial markers CD31 (PECAM) and CD106 (VCAM; see Table 1). However, differences in presence of three markers were found: PL-cultured ASC had significant higher levels of CD73 [mean fluorescence intensity (MF): PL 765 ± 239 vs. FBS 50 ± 12, *p* < 0.05; Fig. 3a], CD90 (MF: PL 3,180 ± 1,065 vs. FBS 206 ± 89, *p* < 0.05; Fig. 3b) and CD166 (MF: PL 164 ± 76 vs. FBS 17 ± 4, *p* < 0.05; Fig. 3c).

**Table 1:** Cell surface marker profile of FBS and PL-cultured ASC

Cell surface marker	FBS	PL
Lin-1	−	−
CD29	++	++
CD31	−	−
CD34	+	+
CD45	−	−
CD54	+	+
CD73	+	++
CD90	++	+++
CD105	++	++
CD106	−	−
CD117	−	−
CD166	+	++
HLA-ABC	++	++
HLA-DR	−	−

Results expressed as mean fluorescence intensity (MF); − MF < 10; + MF 10–100, ++ MF 100–1,000; +++ MF > 1,000, as previously described by Varma et al. (2007)

MF isotype control <8

### Transwell migration

**Fig. 4:**
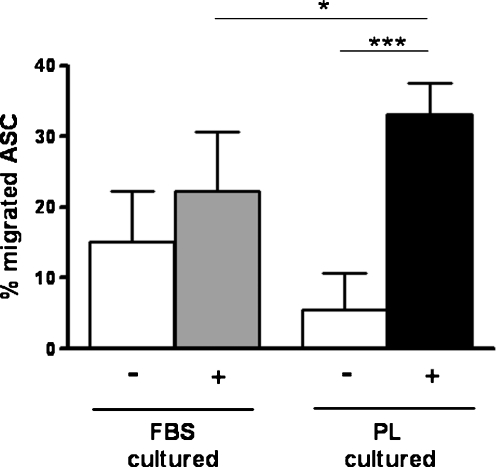
PL-cultured ASC have a higher migration rate than FBS-cultured ASC. Migration of FBS- and PL-cultured ASC towards a stimulus (+; combination of 2% FBS and 1% PL) and without a stimulus (−) were compared. Results are shown as percentage migrated ASC of total ASC applied. *Bars* means ± SD. FBS: *n* = 7; PL: *n* = 6; *p < 0.05

Using a transwell assay, we showed that both FBS- and PL-cultured ASC were capable of migrating towards a stimulus (a combination of 2% FBS and 1% PL in DMEM). FBS-cultured ASC showed 15.1 ± 7.1% migration in the absence of the stimulus, and showed only a 0.5-fold increase in the presence of the stimulus. In contrast, PL-cultured ASC showed 5.6 ± 5.1% migration in the absence of the stimulus, but a significant 5-fold increase (*p* < 0.001) in the presence of the stimulus (Fig. 4). Moreover, significantly more PL-cultured ASC migrated compared with FBS-cultured ASC in the presence of the stimulus (33.2 ± 4.4 vs. 22.3 ± 8.4%, respectively; *p* < 0.05).

### Differentiation towards cardiomyocytes

**Fig. 5:**
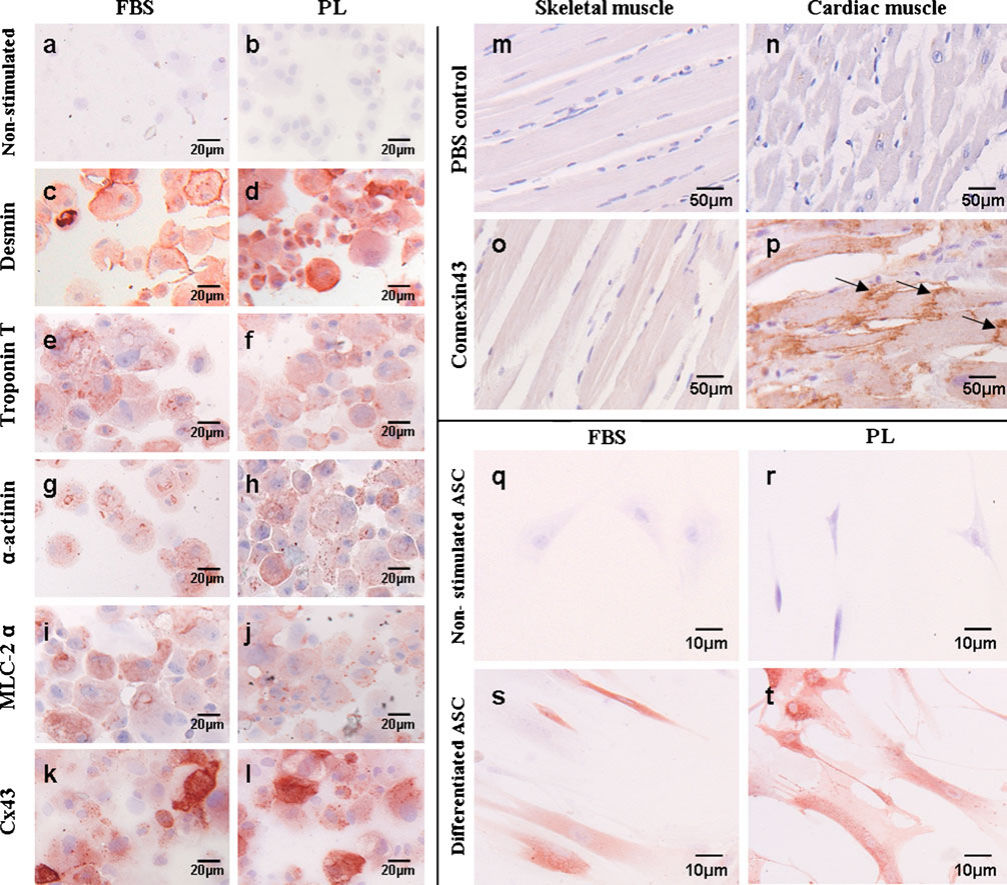
Presence of markers indicative for cardiomyocyte differentiation. **a**–**l** Microscopical images of staining of non-stimulated (**a**, **b**) and 5-aza-2-deoxycytidin stimulated ASC, both FBS- and PL-cultured on cytospin slides for desmin (**c**, **d**), troponin T(**e**, **f**), α-actinin (**g**, **h**), MLC-2α (**i**, **j**) and connexin43(**k**, **l**) at day 10. **m**–**p** Microscopical images of Cx43 staining of paraffin-embedded human adult tissue. Cx43 is present in cardiomyocytes (**p**, *arrows*), not in skeletal muscle (**o**). PBS controls were also negative (**a**, **b**). **q**–**t** Microscopical images of Cx43 staining of ASC on chamber slides. Cx43 is present in 5-aza-2-deoxycytidin stimulated ASC at day 10, both FBS- (**s**) and PL-cultured (**t**), but not in non-differentiated control ASC (**q**–**r**)

**Fig. 6:**
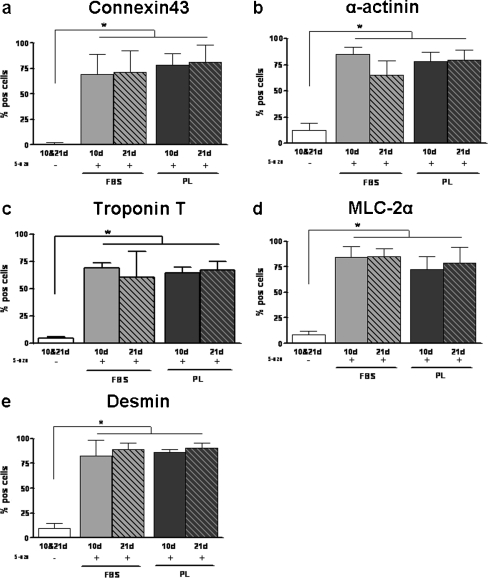
Differentation of FBS- and PL-cultured ASC towards cardiomyocytes. Number of FBS- and PL-cultured ASC positive cells for connexin43 (**a**), α-actinin (**b**), troponin T (**c**), MLC-2α (**d**), and desmin (**e**) when unstimulated (−) or stimulated with 5-aza-2-deoxycytidin ASC at days 10 and 21. Results are compared with non- stimulated ASC. No significant differences in presence of cardiac proteins were found between FBS- and PL-cultured ASC. *Bars* means ± SD. *n* = 6; *p < 0.05

Since PL-cultured ASC differed in several assays from FBS-cultured ASC, we studied whether they were still capable of differentiating towards cardiomyocytes. To determine this, ASC were stimulated with 5-aza-2-deoxycytidin. After 10 and 21 days, the occurrence of different markers present on cardiomyocytes and the microscopical morphology were analyzed. First, we showed that desmin, α-actinin, troponin T, myosin light chain-2α (MLC-2α), and Connexin43 (Cx43) were present in stimulated FBS- and PL-cultured ASC, and not in non-stimulated ASC (Fig. 5a–l). Desmin, α-actinin, troponin T, and MLC-2α are also present on skeletal muscle; however, we demonstrated that Cx43 was only present in human adult cardiomyocytes, and absent in human adult skeletal muscle (Fig. 5m–p). In addition, we found that stimulation also induced morphological changes shown in stimulated ASC in chamberslides that were stained with Cx43: non-stimulated ASC displayed fibroblast morphology, whereas the stimulated ASC, both FBS- and PL-cultured, appeared much larger and more elongated resembling muscle cells (Fig. 5q–t). Furthermore, we quantified the number of positive cells for all markers (Fig. 6a–e). Between 65 and 86% of both FBS- and PL-cultured ASC stained positive for all markers, which was significantly higher compared with non-stimulated ASC (1–14%; *p* < 0.05). No significant differences were found between 10 and 21 days after stimulation, nor between FBS- and PL-cultured ASC, indicating no difference in differentiation capacity (Fig. 6). Notably, no dedifferentiation was found after 12 weeks for either FBS- or PL-cultured ASC (data not shown).

## Discussion

In this study, we demonstrated that culturing ASC in PL-supplemented medium had several favorable effects on in vitro characteristics that will be of importance when ASC are to be used for cardiac repair applications. We found that culturing in PL-supplemented medium increased proliferation rate and enhanced attachment rate, while ASC remained smaller. Although FBS- and PL-cultured ASC had a comparable cell surface marker profile, higher levels of CD73, CD90, and CD166 were found in PL-cultured ASC. PL-cultured ASC also showed an enhanced migration rate. Finally, the differentiation capacity towards cardiomyocytes did not differ between FBS- and PL-cultured ASC.

We found that culturing ASC in PL-supplemented medium significantly increased proliferation rate by 3-fold compared with FBS-cultured ASC in all early passages. A similar proliferation rate of PL-cultured ASC was found by others, suggesting that PL batches are relatively consistent of composition. And in accordance to what has been described by others, PL-cultured ASC could be cultured up to passage 10 without significant growth decline (Blande et al. 2009; Crespo-Diaz et al. 2011; Mirabet et al. 2008). This indicates that, independent of the time point of clinical application of ASC, more cells can be obtained when cultured in PL-supplemented medium compared with FBS-supplemented medium, thus facilitating stem cell therapy.

When ASC are to be injected intravenously, they should be able to attach to the injured endothelium. Therefore, we first analyzed attachment to tissue culture plastic for several successive time points and found that PL-cultured ASC adhered more rapidly than FBS-cultured ASC. The attachment rate of FBS-cultured ASC was comparable with the results of others (Park et al. 2009; van Dijk et al. 2008a), while to the best of our knowledge attachment of PL-cultured ASC has not been determined before. We found no difference in attachment between FBS- and PL-cultured ASC at 24 h. Since ASC seeded in the above-described proliferation assay were washed after 24 h, we can thus conclude that the higher proliferation rate was not caused by the higher attachment rate of PL-cultured ASC. As described above, ASC should also be able to attach to endothelial cells in order to extravasate into the tissue. We indeed demonstrated that ASC were able to attach to primary endothelial cells (HUVEC), and that significantly more PL-cultured ASC adhered after 60 min. Notably, there was a difference in the number of ASC attaching to primary endothelial cells (maximum 50%) compared to tissue culture plastic (maximum 90%) at 60 min. A possible explanation for this finding is that tissue culture plastic is optimized for attachment of cells, and cultured endothelial cells are not. In cases of an acute myocardial infarction, increased attachment of ASC to the injured endothelium might facilitate infiltration of ASC into the infarcted area of the heart, which theoretically would improve stem cell therapy.

As we are interested in intravenous application of ASC after a myocardial infarction, it is important that cells remain as small as possible during in vitro expansion. Therefore, an important advantage of PL-cultured ASC is that they remain a significant 25% smaller (16.0 ± 0.2 μm) compared with FBS-cultured ASC (21.4 ± 0.5 μm), which had never been shown before. Furthermore, PL-cultured ASC have a narrower distribution of cell size, implicating that fewer large cells are present. As mentioned above, cell size is especially important when cells are injected intravenously, since larger cells can lead to obstructions in the lung (Fischer et al. 2009; van Dijk et al. 2011). In addition, it has also been described that smaller stem cells in general could indicate that these cells have a higher potential for cell therapy, such as a higher differentiation capacity (Haasters et al. 2009; Prins et al. 2009; Smith et al. 2004).

Using flow cytometry, we showed that FBS- and PL-cultured ASC had a similar cell surface marker profile, except for significant higher levels of CD73, CD90, and CD166 in PL-cultured ASC. Other studies also compared the surface marker profile of FBS- and PL-cultured ASC but found no differences (Blande et al. 2009; Crespo-Diaz et al. 2011; Kocaoemer et al. 2007; Shih et al. 2010). Notably, these studies analyzed high passage ASC (p4–7) while we analyzed early passage ASC (p2). We focused on early passage ASC, since these cells have a higher therapeutic potential compared with high passage ASC (Zhu et al. 2008). It has been shown that levels of CD73, CD90, and CD166 increase upon passaging of cultured ASC, which could explain the differences found in surface markers (Varma et al. 2007; Shih et al. 2010). These surface markers are described as mediators of cell adhesion and migration, for example in transendothelial cell migration of leukocytes (Davies et al. 2008; Rege and Hagood 2006; Swart et al. 2005; Colgan et al. 2006).

When ASC are applied intravenously, it is important that, after initial attachment, ASC are capable of migrating into the injured tissue. To provide a first indication of the migration capacity of FBS- and PL-cultured ASC, transwell experiments were performed using serum as a stimulus for migration . We are the first to show that PL-cultured ASC had a significantly higher migration rate compared with FBS-cultured ASC. Although more experiments are needed using specific chemokines, this finding is a first indication that ASC are capable of migrating towards a stimulus.

Finally, we investigated whether the fast proliferating PL-cultured ASC were still capable of differentiating towards cardiomyocytes. We stimulated ASC with 5-aza-2-deoxycitidin, which is described in the literature as inducing differentiation towards cardiomyocytes in various stem cells (Xu et al. 2002; Hakuno et al. 2002; van Dijk et al. 2008b). Full in vitro differentiation of human ASC into functional beating cardiomyocytes without co-culturing has to our knowledge never been shown. However, we and others have shown that in vitro ASC are capable of presenting markers indicative for differentiation towards cardiomyocytes (Gaustad et al. 2004; Crespo-Diaz et al. 2011; van Dijk et al. 2008b), although whether these markers are cardiomyocyte-specific is debated. In line with our previous research (van Dijk et al. 2008b), we showed that desmin, troponin T, α-actinin, and MLC-2α, were present in both FBS- and PL-cultured stimulated ASC. Furthermore,we have now also shown that Cx43 was also present in both FBS- and PL-cultured stimulated ASC. Since electrical coupling between heart muscle cells is dependent on Cx43-rich gap junctions, the presence of this protein is an important feature of differentiation towards cardiomyocytes (Beyer et al. 1987). Additionally, we demonstrated that Cx43 was only present in adult cardiac tissue and not in adult skeletal muscle tissue, indicating specificity for adult cardiac muscle cells. Moreover, we found that the morphology of both FBS- and PL-cultured ASC changed after stimulation: ASC appeared much larger and more elongated, resembling muscle cells. Furthermore, we quantified the number of positive cells for all markers, and demonstrated that there was no difference in the presence of all markers between FBS- and PL-cultured ASC, nor between 10 and 21 days after stimulation. This therefore indicates that both FBS- and PL-cultured ASC started differentiating towards cardiomyocytes. Previously, van Dijk et al. (2008b) described that 5-aza-2-deoxycitidin stimulation was effective in differentiating FBS-cultured ASC towards cardiomyocytes (minimal 28% positive cells for α-actinin to maximal 51% for desmin. Interestingly, now we found more positive cells (minimal of 65% for α-actinin to maximal 91% for desmin), both for FBS- and PL-cultured ASC. This might be explained by lower passage ASC that were used in this particular study (p2 vs. p3–5 by van Dijk et al. 2008b). As such, in a low passage, more ASC appear to be able to differentiate towards cardiomyocytes, and are therefore more favorable for therapeutic purposes in the heart. Only one other study has shown that, in 5-aza-2-deoxycytidin stimulated PL-cultured ASC, the markers troponin T and MLC-2α were present; however, the authors did not quantify their number of positive cells and did not study other markers (Crespo-Diaz et al. 2011).

To summarize, culturing ASC in PL-supplemented medium has multiple beneficial effects compared with FBS-supplemented medium that are important when these ASC are applied to facilitate cardiac repair: (1) increased proliferation rate, resulting in a large number of ASC in a short culture period, without affecting the capacity to differentiate towards cardiomyocytes; (2) smaller cell size, which theoretically reduces the chance of vascular obstruction when applied via intravenous injection; and (3) faster attachment and migration rate, important for attachment and extravasation of ASC at the site of injury. Finally, PL can be directly implemented for clinical applications since it is GMP approved. In conclusion, our study indicates that culturing ASC in PL supplemented medium is more favorable compared with FBS when ASC are to be applied as cell therapy, for example in patients with myocardial infarction.

## Abbreviations

ASC: Adipose-derived stromal cell
FBS: Fetal bovine serum
PL: Platelet lysate
MF: Mean fluorescence intensity
